# Fundamentals of Care in a 1997 Azorean Disaster: A Multiple-Case Study

**DOI:** 10.3390/nursrep16030089

**Published:** 2026-03-05

**Authors:** Eunice Gatinho Pires, Cristina Lavareda Baixinho, Adriana Henriques, Andreia Costa

**Affiliations:** 1 Nursing Research Innovation and Development Centre of Lisbon (CIDNUR), Escola Superior de Enfermagem (ESEUL), Universidade de Lisboa, 1600-096 Lisboa, Portugal; crbaixinho@esel.pt (C.L.B.); ahenriques@esel.pt (A.H.); andreia.costa@esel.pt (A.C.); 2 Hospital do Divino Espírito Santo—EPER, 9500-370 Ponta Delgada, Portugal; 3 Instituto de Saúde Ambiental (ISAMB), Faculdade de Medicina, Universidade de Lisboa, 1649-028 Lisboa, Portugal; 4 Center for Innovative Care and Health Technology (ciTechcare), 2414-016 Leiria, Portugal; 5 Laboratório Associado TERRA, Faculdade de Medicina, Universidade de Lisboa, 1649-028 Lisboa, Portugal

**Keywords:** disaster nursing, patient-centred care, fundamental care, case reports, qualitative research

## Abstract

**Background/Objectives**: Disasters have a substantial impact on health systems and populations worldwide, with increasing frequency, mortality, and economic losses associated with natural hazards. The United Nations emphasises that disasters result from the interaction between hazards, exposure, and vulnerability, requiring integrated, people-centred health responses aligned with the 2030 Agenda. However, empirical evidence describing specific nursing interventions, particularly during response and recovery phases, is limited. This study aims to analyse the fundamental nursing care interventions provided to disaster victims in the Autonomous Region of Azores, Portugal. **Methods**: A qualitative multiple case study was conducted using documentary analysis of the nursing records from two disaster survivors with different clinical trajectories. Data were collected between August 2023 and May 2024 through complete transcription of nursing documentation contained in the clinical files. Data analysis followed Yin’s case study methodology and was theoretically supported by the Fundamentals of Care Framework. **Results:** The findings indicated a predominance of interventions addressing physiological needs during the acute phase, which progressively evolved to maintenance, psychosocial support, and adaptation needs during prolonged hospitalizations. Nursing care integrates advanced technical skills with relational and person-centred interventions, including emotional support, therapeutic communication, and promotion of patient autonomy. **Conclusions**: Nursing practice in disaster situations should be conceptualised as integrative, person-centred care grounded in international nursing frameworks. Strengthening disaster-specific nursing education, developing phase-adapted care protocols, and promoting multicentre longitudinal research appear to play a critical role for advancing nursing care models and informing health policies in disaster-prone regions.

## 1. Introduction

Disasters are unpredictable by nature. Affect health systems and populations globally: social, economic, and racial groups without warning and can last from hours to months [1]. The last Disasters in Numbers report confirms this trend, with 393 disasters associated with natural hazards in 2024, accounting for 16,753 deaths and economic losses exceeding £241.95 billion [2].

In 2024, several extreme events worldwide highlighted the increasing impact of natural disasters. Extreme temperature phenomena in Asia, severe droughts in Africa, and landslides in Papua New Guinea have resulted in major humanitarian crises that are considered among the most serious ever recorded in these territories. In Japan, a high-intensity earthquake caused 551 deaths and was one of the ten most costly natural disasters in 2024, with economic losses estimated at approximately $15 billion. Similarly, in Spain, floods in the Valencia region were among the ten most costly disasters worldwide, with losses estimated at approximately 11 billion dollars. The United States experienced significant impacts associated with multiple extreme weather events throughout 2024, including large-scale hurricanes such as Helene, Milton, and Beryl, which stand out among the most costly natural events in the country’s recent history [2].

The United Nations considers that disasters are not natural or inevitable phenomena but result from the interaction of risk events with high levels of vulnerability and exposure. Therefore, their prevention or mitigation requires concrete human action [3].

The document drawn up by the United Nations, consisting of 17 Sustainable Development Goals, is a broad agenda that addresses various dimensions of sustainable development (social, economic, and environmental) and promotes peace, justice, and effective institutions. One goal is to make cities and communities inclusive, safe, resilient, and sustainable. By 2030, the number of deaths and people affected by disasters should be significantly reduced, as well as direct economic losses, with a focus on protecting the vulnerable populations and communities [4].

In collaboration with the World Health Organisation (WHO), the International Council of Nurses (ICN) recognises Disaster Nursing as a specialised area defined as systematic nursing care based on specific knowledge and techniques to reduce risks and mitigate the health impacts of disaster events [5]. The ICN emphasises that nurses need extensive training in disaster medicine and management and proposes a competency framework structured around four phases of the disaster cycle: prevention, preparedness, response, and recovery [5].

The framework identifies three levels of competence: Level I covers the essential competencies that all nurses should possess to ensure minimum disaster preparedness. Level II is intended for nurses trained to participate in institutional or organisational disaster responses. Level III is intended for experienced professionals in specialised teams to manage highly complex scenarios [5].

The competencies are organised into eight domains: preparation and planning (readiness and confidence for action in the event of a disaster), communication (information flow and decision-making support), incident management systems (implementation of coordinated response structures), safety and security (safe practices to prevent further harm), assessment (gathering data on patients, families, and communities to guide care), intervention (triggering appropriate responses within incident systems), recovery (measures to restore or increase resilience at the individual, family, or community level), and law and ethics (regulatory and ethical frameworks for nursing practice). This framework demonstrates that disaster nursing goes beyond clinical intervention, encompassing planning, coordination, communication, and leadership, and positions nursing as a strategic element in building resilient health systems [5].

Although disaster nursing has evolved significantly in terms of competencies and operational preparedness, reviews indicate that its theoretical consolidation within nursing science remains fragmented, with limited integration of discipline-specific conceptual frameworks to systematically guide clinical practice [5,6].

Scientific production remains largely focused on the acute phase of hospital response and the assessment of self-perceived competencies, presenting limited theoretical operationalisation of care throughout the disaster continuum, particularly in the processes of recovery, adaptation, and rehabilitation in the medium and long term [7]. This conceptual weakness, combined with a lack of primary research, makes it difficult to define, operationalise, and evaluate nursing interventions in the context of disasters [7]. Simultaneously, nursing care in disaster situations occurs in highly complex and unpredictable contexts with limited resources and intense pressure to provide care, thus requiring integrative models that guide rapid decisions and consistent care [7,8].

The absence of a guiding theoretical model can lead to fragmented and inconsistent practices, making it difficult to systematise care and guarantee quality. Thus, nursing interventions in disasters must be supported by a solid theoretical model that allows for the structuring of clinical thinking, supports the prioritisation of interventions, and ensures a coherent, ethical, person-centred response, even in highly adverse scenarios. Thus, the Framework of Fundamental Care (FoC) is a valuable tool for guiding nursing care in disaster contexts [9]. By integrating the fundamental needs of the person, the nurse–client relationship, and the context in which care is provided, this framework offers a holistic and flexible approach that is particularly relevant in crisis situations. Its application can help ensure that, even in disaster scenarios, nursing care remains focused on human dignity, safety, and the overall well-being of those affected, promoting consistent, humanised, and theoretically grounded interventions.

The FoC emphasises the ability of nurses to establish a therapeutic relationship with patients, enabling their basic needs to be met, and focusing on the assessment, planning, implementation, and monitoring of care around fundamental needs [10]. The specific contribution of nursing is to ensure that these needs are met in a competent, respectful, and empathetic manner, supported by three elements: trust-based relationships, integration of care needs, and system-level commitment to creating favourable conditions [9,10,11,12]. In this context, assessing and addressing patients’ and caregivers’ needs is essential to ensure safety, well-being, and recovery, requiring individualised, compassionate, and clinically effective care [11,13].

This study addresses a specific gap in disaster nursing scholarship: despite calls for stronger theoretical grounding, little is known about how fundamental nursing care is documented and integrated in real-world disaster-related hospital admissions, particularly beyond the immediate acute phase. Moreover, evidence using the FoC Framework remains fragmented, and rarely combined with standardised nursing terminologies to interrogate clinical records. By conducting a retrospective multiple-case documentary analysis of two disaster-related admissions (1997), this study offers an original contribution through (i) a historically grounded reconstruction of nursing documentation across acute and early recovery phases, and (ii) a theoretically informed mapping of documented nursing practices to both the FoC Framework and International Classification for Nursing Practice (ICNP), making the nursing contribution analytically visible and transferable through analytic generalisation. Therefore, this study aimed to analyse the fundamental nursing care interventions provided in disaster situations in the Autonomous Region of the Azores.

## 2. Materials and Methods

### 2.1. Study Design

A multiple case study was conducted, allowing for the intensive research necessary to clarify complex and little-known phenomena.

This design restricted the inquiry to a small number of cases in which subunits of analysis could be observed and using multiple sources of evidence. Furthermore, promoting the analysis of phenomena in their real contexts, it allows for the investigation of their limits and their relationship with the different scenarios in which they occur [13]. This was a qualitative, exploratory, retrospective, multiple-case study based on documentary analysis of clinical records and Yin’s theoretical replication logic [13], which included inter-case and cross-case analyses.

Specifically, replication was guided by a priori theoretical propositions derived from the FoC framework. These propositions included the following: (1) that disaster-related nursing documentation would reflect integration of physical, psychosocial, and relational dimensions of care; (2) that variations in clinical trajectory (acute critical versus prolonged recovery) would produce distinct yet theoretically coherent patterns of fundamental care delivery. Literal replication was achieved by applying the same analytical protocol to both cases, while theoretical replication was operationalised through the intentional selection of contrasting care trajectories to examine whether the proposed theoretical patterns were confirmed under different clinical conditions.

**Figure 1:**
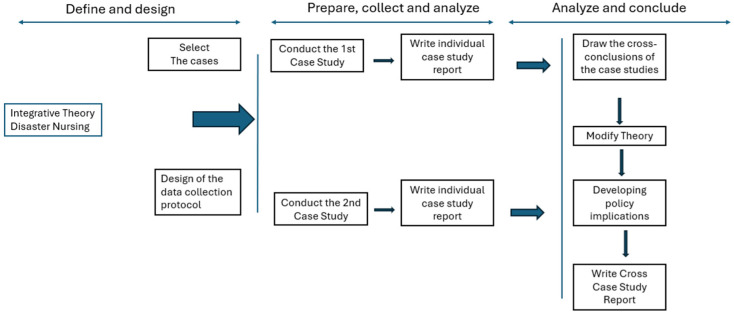
Preliminary case study protocol, adapted from Yin, R.K. (2018) [13].

According to the author, a case study is an empirical method that investigates a contemporary phenomenon (the ‘case’) in depth and in its real-world context, especially when the boundaries between the phenomenon and context may not be clearly evident [13]. The author advocates the use of a case study protocol (Figure 1), as it enables the systematic collection of evidence through a predefined set of substantive questions and clearly specifies the procedures and general rules guiding its application [13].

The development of a case study protocol is recommended under all circumstances and is considered essential when conducting multiple-case studies. According to the same author, the protocol requires anticipation of potential methodological challenges, including those related to the completion and presentation of case study reports [13]. Preparing this report before data collection was important because it allowed for planning at this stage, which is included in the arrow between ‘prepare’ and ‘analyse’ in Figure 1. Although presented sequentially in Figure 1, the “prepare” and “analyse” phases were operationalised iteratively. Data preparation (including extraction, transcription, and organisation) informed initial analytical insights, which in turn guided further refinement of data structuring and categorisation. This cyclical interaction ensured analytical coherence and methodological transparency throughout the study. The manuscript was developed in accordance with the Standards for Reporting Qualitative Research (SRQR) guideline, and the corresponding SRQR checklist was used during the editing process [14].

Given the knowledge gap in the area of response and recovery, this study examined how basic human needs manifested in survivors hospitalised after the 1997 Azores disaster and what nursing interventions were applied, guided by the FoC and the International Council of Nurses’ reference frameworks. Although the disaster occurred in 1997, its relevance for this study is analytical rather than chronological. The historical records provide a rare opportunity to examine longitudinal documentation of nursing care across acute and recovery phases in a disaster context. By applying a contemporary theoretical lens to this documentation, the study contributes to understanding enduring dimensions of fundamental nursing care, while recognising that transferability to current systems depends on contextual similarity rather than temporal equivalence.

This study addressed the following aspects: (i) which basic needs were most compromised, (ii) which nursing interventions were used, and (iii) similarities and differences between cases. The objectives were to identify compromised needs, analyse nursing interventions during hospitalisation, compare cases to highlight patterns, and analyse the implications for developing nursing care models in disaster situations.

### 2.2. Setting

In the Azores, collective memory is marked by extreme natural phenomena, such as volcanic eruptions, earthquakes, floods, storms, and landslides, which have caused significant human and material losses over the centuries [15,16]. The geographical isolation of this region in the middle of the Atlantic, its territorial fragmentation, and scarcity of resources accentuate the vulnerability of its populations [16]. In particular, landslides, often associated with intense or prolonged rainfall, deforestation, and disorderly urbanisation processes, account for a substantial proportion of natural disasters recorded globally and are responsible for more than 40% of events reported annually [2,6].

On 31 October 1997, a major landslide occurred in a remote parish in the Azores, resulting in the death of 25 people and profound and lasting impacts on survivors, with physical, psychological, social, and economic repercussions lasting beyond the initial event. Some victims remain buried for long periods, experiencing increased suffering due to the death of close family members in the same space [15].

### 2.3. Participants

Two cases were selected that met the eligibility criteria (Table 1). Case selection followed purposive, theoretically informed criteria within the constraints of retrospective record availability. From the eligible disaster-related admissions identified, we included cases presenting (a) verified disaster exposure with subsequent hospital admission, and (b) nursing documentation covering both acute stabilisation and inpatient care, enabling analysis of care integration over time. Encounters limited to the emergency department with discharge within <24 h were excluded because they did not provide sufficient longitudinal documentation to examine the FoC dimensions. The two selected cases supported a multiple-case logic: literal replication (same analytic protocol applied to both cases) and theoretical replication (contrasting care trajectories across Intensive Care Unit (ICU) versus orthopaedic prolonged recovery).

### 2.4. Data Collection

Data collection was carried out by consulting the hospital’s clinical records, from which the records were verbatim extracted and transcribed manually, in full, between August 2023 and May 2024, after prior approval by the institution’s Ethics Committee. All physical clinical records between 31 October and 11 November 1997 were consulted, looking only at the reason for admission, for a total of 31 records. Six records that met the eligibility criteria upon initial consultation with the clinical records regarding the reason for admission were identified. After this consultation, the six cases identified at admission, four involved patients who were treated exclusively in the Emergency Department for minor injuries, with a length of stay of less than 24 h. These cases contained only medical records and did not include any nursing documentation. In contrast, two clinical records had nursing records for hospitalisation with nursing interventions, one in an intensive care unit (case 1) and one in an orthopaedic unit (case 2). After selecting these two clinical cases, the records were manually transcribed verbatim into a Word document, and all identifiable information was removed during transcription to ensure anonymisation at this stage of data handling. Subsequently, the research team created a case study database consisting of a formal set of evidence distinct from the final case study report, containing all case study notes, documents, and preliminary narratives about the data.

### 2.5. Data Analysis

Data analysis followed a theory-informed qualitative documentary approach. Extracted nursing records were examined systematically, and discrete documented statements, including clinical observations, recorded interventions, and relational or contextual notes, were treated as the units of analysis. A structured analytical matrix was developed a priori based on the three dimensions of the FoC framework—relational, physical, and psychosocial—to guide deductive categorisation. Within each dimension, documentary excerpts were organised, compared, and iteratively refined to identify recurrent patterns, contextual variations, and meaningful configurations of care.

Each case was first analysed independently using this structured matrix to produce an in-depth case description. Subsequently, a joint display matrix was constructed to facilitate cross-case comparison, enabling systematic pattern matching and identification of convergences, divergences, and theoretically relevant variations in the integration of fundamental care across contrasting clinical trajectories. This stepwise analytic process enhanced conceptual coherence and supported analytic generalisation.

The primary analysis was conducted by the principal investigator using the structured analytical matrix. To strengthen credibility and confirmability, selected excerpts and their categorisation were independently reviewed by the supervisory team. Interpretative differences were examined in structured review meetings until consensus was achieved. Although full double coding of the entire dataset was not undertaken, this peer-review and consensus-based validation process, supported by an audit trail documenting analytic decisions and matrix refinements, reinforced the transparency, dependability, and methodological rigour of the analysis.

These strategies were important for practising the study analysis technique through pattern matching, explanation building, cross-data synthesis, and logical model construction. The nursing documentation produced in 1997 was predominantly narrative and paper-based, with varying degrees of structuring depending on the unit. The ICNP for Disaster Nursing was applied as an existing standardised classification system [17]. Mapping followed a stepwise approach: (1) verbatim extraction and transcription of nursing notes; (2) identification of discrete nursing actions, observations, and relational/psychosocial statements; (3) categorization under the FoC dimensions; (4) ICNP for Disaster Nursing concept mapping using the 2017 version, with a coding dictionary developed a priori and iteratively refined. The combined use of these references allowed for more comprehensive, systematic, and comparable analyses of nursing care, thus reinforcing the validity and transferability of the study results. Coding was performed by author E.G.P., with author C.L.B. independently reviewing of the coded material. Discrepancies were discussed until consensus; unresolved cases were adjudicated by authors A.C. and A.H. All coding decisions and changes to the coding dictionary were recorded to maintain an audit trail.

### 2.6. Techniques to Enhance Trustworthiness

Methodological rigour and reliability were ensured through a historical design based on two theory-driven case studies conducted in accordance with the established principles of case study research [13].

The selection included those who, in the reason for admission, were referred to as ‘victim of catastrophe’, ‘buried victim’, or “a victim presenting with multiple traumatic injuries secondary to burial” who was admitted after observation in the Emergency Department. Data collection and analysis followed a replication logic, allowing for literal and theoretical replication between cases. Each case represents a complete unit of analysis. Individual case analyses and a synthesis between cases were performed to examine the converging and contrasting evidence related to the study’s propositions, thus increasing analytical transparency.

Given the single primary data source (clinical records), we used theoretical triangulation FoC [9] and ICNP [17] as well as multiple-case pattern matching. A clear chain of evidence was maintained through a dedicated case study database containing original documents, full transcripts, and analytical matrices. To enhance analytical credibility, a structured peer debriefing process was undertaken. A purposive sample of extracted documentary excerpts and their categorisation within the analytical matrix was independently reviewed by an experienced qualitative researcher. The review assessed consistency between the original clinical records, the extracted data, and the assigned conceptual categories. Observations and potential discrepancies were discussed with the research team, and refinements were made by consensus. This process functioned as peer debriefing.

Internal validity was addressed through pattern matching and explanation building, operationalized by comparing documentary evidence against a priori propositions derived from FoC (e.g., whether nursing documentation evidence integrated physical, psychosocial, and relational care), first within each case and then across cases. External validity was achieved through analytical generalisation based on theoretical replication across two contrasting clinical trajectories [18].

Reliability was ensured by adhering to a formal case study protocol and standardised procedures for data extraction, transcription, and analysis. All study materials were systematically archived to support the verification and reproducibility of the results [18].

## 3. Results

### 3.1. Characterisation of Cases

- Case Study 1

The landslide occurred during the early hours of the morning, and the victims were buried under mud and debris, such as tree trunks, for a long period of time until they were found by neighbours. According to data extracted from the hospital’s clinical record (hereafter referred to as the P), the patient was admitted to the Emergency Department (ED) with the following diagnoses: cerebral anoxia, cortical blindness, acute ischaemic lesions in the bilateral occipital lobes, haemothorax on the right, extensive pleural effusion on the right, tachycardia, hypotension, polypnea, ecchymosis, abrasions, and vomiting. Due to severe chest trauma associated with cerebral anoxia, the patient required admission to the ICU, followed by continued hospitalisation in an inpatient unit (ED: 1 d, ICU: 5 days, Inpatient Unit: 9 days, total: 15 days).

- Case Study 2

According to the data extracted from P, the victim was admitted to the ED after being buried alive, with multiple traumas and hip fractures. She was initially observed in the ED and underwent surgery to fix her pelvis, followed by admission to the Orthopaedic Trauma Department for 45 days after the injury.

### 3.2. Contextualisation of the Cases

Both cases involved the same disaster, a landslide that buried several families and killed 25 people. In 1997, there were no nearby hospitals or health facilities; therefore, the victims were evacuated after receiving initial medical treatment at the scene several hours after the event and were transported by helicopter to the only hospital on the island, located approximately 80 km away. Although they were victims of the same natural disaster (landslides), their hospitalisations resulted from different diagnoses.

Nursing records extracted from the Nursing Documentation (D) were based on the assessment of the activities of daily living performed by the patient with or without assistance, identification of needs and consisted of the following descriptions: general assessment of the patient: awake, sleepy, calm, agitated, and anxious; hygiene and comfort care: cooperative, uncooperative, independent, or with assistance; serum therapy in peripheral vein or central venous catheter: permeable or non-permeable; prescribed therapy: complied, did not comply, change of therapy; pain: present, absent, therapy prescribed in SOS; Feeding: good, poor, quantity; skin integrity: yes, no, care provided autonomously and/or interdependently; elimination: yes, no, medical device assistance; visits from family/friends; and record of requests for complementary diagnostic and therapeutic resources.

### 3.3. Identification of Domains, Nursing Diagnoses/Interventions, Expected Outcomes and Assessment

The ICNP for disaster nursing uses terminology—nursing diagnoses, outcomes, and interventions—which is most likely to be useful in this context [17]. Data collection using standardised terminology was used to evaluate nursing practice and patient and family outcomes using a set of clinical data, thus making the processes and outcomes of nursing care in disaster situations more visible [17]. The analysis of nursing interventions in both cases revealed distinct disaster response patterns. This shows a clear prioritisation of care based on clinical severity, stage of evolution, and care context (Table 2).

The analysis of the table shows that, in both cases, nursing records were organised by clinical domains and results-oriented, allowing interventions to be related to expected outcomes according to the ICNP [17] and their respective assessments.

In Case 1, the interventions were concentrated in the respiratory and cardiovascular domains, reflecting the priority given to physiological stabilisation in a critical context. The expected outcomes, “effective ventilation and adequate saturation” and “effective perfusion and haemodynamic stability”, showed consistent assessments, with records confirming respiratory and haemodynamic stabilisation. In the pain/trauma domain, analgesia and pain monitoring are associated with an objective improvement in comfort, as reflected by the ability to rest after symptomatic control. In terms of integument/safety, the prevention of pressure injuries and CVC monitoring were aligned with the maintenance of skin integrity and the absence of complications associated with the device, suggesting preventive effectiveness. In the psychological domain, the presence of family and psychological support is associated with reduced crying and greater calmness, indicating measurable emotional gain.

In Case 2, the records emphasised pain/trauma, skin integrity, and mobility/musculoskeletal domains, consistent with prolonged hospitalisation and a high risk of complications from immobilisation.

The expected results indicate effective pain control after therapeutic adjustments, maintenance of protected skin, prevention of pressure ulcers, and preservation of mobility without aggravation. In addition, therapeutic management (including transfusion) resulted in positive outcomes, with therapeutic compliance without incidents. In contrast, the psychological domain showed a less robust result (“partial improvement”), suggesting persistent emotional vulnerability despite the psychiatric intervention.

### 3.4. Analysis of Cases in Light of the Framework of Fundamental Care

In the first phase, the results are presented according to the FoC, which allows the identification and comparison of nursing interventions according to the essential care needs addressed in each case, integrating the dimensions of Relationship, Care Integration and Care Context.

The nursing records in Case 1 showed a strong emotional charge and relational bond after five days of hospitalisation. The patient called the nurses “godmothers”, showed fear of loneliness, and refused contact with the outside world. The interventions included anticipating emotional needs, “calling a family member in case of agitation”, and comfort strategies through emotional bonding. This dimension demonstrates that, in the context of a disaster, the therapeutic relationship is central to emotional balance and acceptance of care.

In Case 2, the records were more focused on objective aspects, such as feeding, collaboration with care, dissatisfaction with food, and response to family visits. No records regarding the trust or empathic involvement of the team were found. This suggests that in prolonged hospitalisation, the nurse–patient relationship was recorded functionally, without highlighting deeper relational aspects (Table 3).

Care Integration, Dimension 2, focuses on the articulation of the physical, psychosocial, and relational foundations of care, reflecting how nursing interventions respond in an integrated manner to the fundamental needs of the person being cared for. This dimension allows us to analyse not only what has been done but also how care has been organised and documented according to the different spheres of care.

Table 4 list the physical fundamentals in Case 1, which are the records extracted in detail D “partial hygiene with little cooperation, reduced food intake, restless sleep, need for analgesia and strict safety monitoring”, “chest drainage, central venous catheter and risk of injury”. These findings demonstrate the importance of life support and immediate comfort. In Case 2, “bedside hygiene for long periods, initial feeding difficulties, sleep regulated with benzodiazepines prescribed by the doctor, prolonged pain control and prevention of pressure ulcers” were observed. This pattern reflects the need to maintain physical well-being and prevent complications during prolonged hospitalisations. All of these dimensions must be considered when working with patients with complex health and care needs, as in a disaster [10].

The psychosocial factors are presented in Table 4. In Case 1, the patient had a “distant look”, avoided contact, expressed fear of being alone, and “cried during visits”, but improved with the presence of family members and the psychologist. This may indicate psychological vulnerability and dependence on emotional support from others.

In Case 2, the patient revealed a “sad expression, discouragement, and depression, requiring psychiatric support”. These records suggest that significant psychological distress may be associated with the trauma experienced during the disaster, functional limitations, and prolonged hospitalisation.

Regarding relational foundations, in Case 1, there were multiple records of empathy, family support, and significant presence of a family member, which were integrated as *coping* resources. In Case 2, relational records were not identified in the nursing documentation.

Dimension 3: The Context of Care, presented in Table 5, refers to institutional policies and systems that provide conditions for care. The third dimension is the contextual factors of care. These factors are divided into two levels: systems and policies. These dimensions influence the responses to a person’s needs and the relationships between them, their families, and nurses.

In the context of care, In Case 1, the institutional policy of restricting the presence of family members at night, “he was very tearful, hugging his family member and saying he didn’t want them to leave”, contrasts with the patient’s evident emotional need for family support. Hospital policy limits the continuity of the emotional bond, showing the tension between institutional norms and individual needs during disasters.

In Case 2, the records indicate that access to mental health support was activated following medical prescription, as reflected in the notes “patient remains in the same condition, sometimes quite discouraged, trying to react” and “doctor requested psychiatric support”. The documentation suggests that referral to specialised psychosocial support occurred through established medical channels. While this may reflect the organisational protocols in place at the time, the available records do not allow definitive conclusions regarding institutional barriers or alternative referral pathways. Therefore, this interpretation should be understood as a contextual observation based on documented activation processes rather than direct evidence of structural constraint.

**Figure 2:**
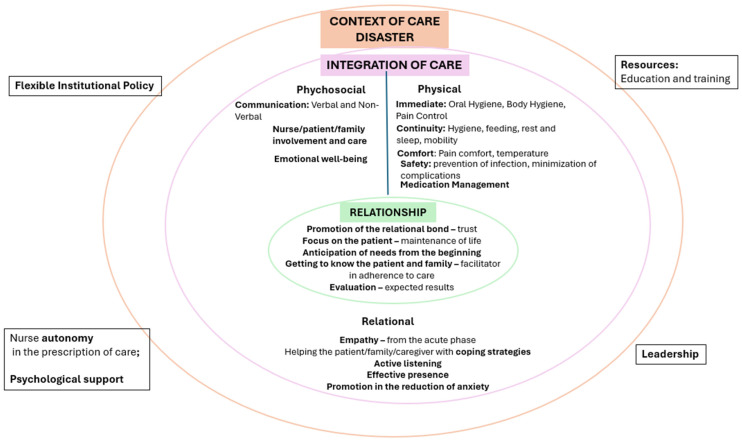
Fundamental of Care Framework for Disasters, adapted from Kitson [9].

While the documentation indicates that specialised psychosocial support was initiated through medical prescription, the records do not allow determination of whether this procedural pathway affected recovery trajectory or length of stay. Any association between referral timing and hospitalisation outcomes should therefore be interpreted cautiously. Based on these observations, several theoretical propositions can be derived:

(a) Nursing practice during disasters is organised into two complementary phases: immediate stabilisation (physiological) and prolonged support (psychosocial and relational).
(b) The effectiveness of care depends on a multidimensional and integrated approach that coherently articulates the dimensions of relationship, care integration, and care context as showed in Figure 2.

## 4. Discussion

It is important to distinguish between findings directly supported by empirical data and the broader theoretical or practice implications derived from their interpretation. Data-grounded findings refer exclusively to nursing needs and interventions explicitly documented in the clinical records. In this multiple-case study, the relational dimension was most evident in Case 1, in which nurses demonstrated concern for care and developed a relationship of trust through their records, thereby achieving a degree of familiarity in which emotional bonds were essential to well-being and recovery. This finding is consistent with evidence describing nurses who demonstrate exceptional skills in disaster situations, effective communication, stress management, problem-solving techniques, empathy, patience, tolerance, and understanding, promoting cooperation, respect, and trust between patients and nurses [19,20]. Once trust was established, the nurse was able to focus on the patient (and their family) by being present with active listening, communicating about fears and emotions, reducing anxiety/crying, and anticipating needs so that the patient felt safe (physical, psychological, and emotional), as recommended by the FoC.

Pene, Aspinall [21] described that although this care may be a favourable time to create a bond and respond to broader needs, this period is often used by nurses to perform their tasks, representing a missed opportunity for therapeutic contact. Although there is a reference to emotional distress in Case 2, no relational interventions are recorded in the available notes. The absence of documented relational care should not be interpreted as evidence that such care did not occur; rather, it reflects a lack of explicit recording. It may be hypothesised that limited documentation of relational engagement could influence the visibility of therapeutic interactions within the care process. However, the present study does not provide comparative data to establish a causal relationship between relational documentation and recovery trajectory or length of hospitalisation. Evidence from the broader nursing literature suggests that therapeutic relationships are associated with perceived quality of care [22,23], but this association cannot be directly inferred from the current dataset.

In the area of integrated care, physical care predominated in critical contexts, focusing on safety and life stabilisation. The results showed that in disaster situations, immediate physiological needs, namely breathing, circulation, elimination, and safety, are prioritised in cases of severe trauma [8,24].

After hospital admission, the most frequently identified needs were related to breathing (oxygen therapy and chest drainage), circulation and fluid balance (fluid therapy, peripheral catheterisation, and urinary output monitoring), and body temperature regulation. Subsequently, the need for psychosocial support was highlighted. These findings are in line with those of other authors who classified “immediate care” as a central dimension of nursing practice in disaster contexts, emphasising that the provision of advanced care at the scene of the event can contribute to reducing preventable mortality [25,26]. However, some authors argue that aspects such as mobility, rest and sleep, hygiene, and nutrition have a more comprehensive impact on the patient’s health and well-being, influencing nutritional status, occurrence of delirium, emotional well-being, and satisfaction with the care provided [10].

The integration of the physical and psychosocial components of care tends to be associated with more consistent results. In the present study, this was evidenced by the coexistence of interventions aimed at clinical stabilisation and comfort with emotional support and family involvement, contributing to simultaneous outcomes, namely, greater clinical stability, symptomatic relief, tranquillity, and, consequently, greater patient adherence to the care plan. This assumption is consistent with the literature, which describes how experienced clinical nurses actively promote patient and family involvement, as well as coordination with the nursing team and the wider interprofessional team, discussing care needs, and gathering real-time feedback on the essential care provided [21]. This practice has been associated with benefits, including improvements in patient involvement and perceived quality of care [10,21,22].

A key analytical finding concerns the differentiation of adaptive and relational needs across the two cases when examined comparatively. In Case 1, documentation predominantly reflected physiological instability requiring intensive monitoring and technological support (oxygenation, fluid therapy, and chest drainage) with relational elements minimally recorded during the acute phase. In contrast, in Case 2, demonstrated greater visibility of psychosocial distress, including expressions of sadness, discouragement, and documented requests for psychiatric support, while physical stability allowed for a broader emergence of adaptive and emotional needs. This contrast suggests that the integration and visibility of relational and psychosocial dimensions of care may vary according to clinical trajectory and phase of recovery. Rather than representing isolated phenomena, the two cases illustrate how the balance between physical stabilisation and adaptive support shifts over time in disaster-related hospitalisation. This comparative pattern aligns with literature emphasising the growing centrality of mental health and adaptation processes in post-disaster recovery phases [27].

The authors have identified that disasters have a negative psychological impact on survivors, making “psychosocial care” crucial, which is why it is essential to offer psychological support and care to victims during and after a catastrophic event [23,28]. Survivors may experience fear, guilt, and anger in such situations. They may constantly relive the event in their minds and dreams, avoid stimuli that remind them of the event, and have trouble sleeping [19,25]. Empathy for victims’ reactions to stress and pain is essential so that they feel heard and supported, helping them adapt to traumatic situations and become resilient.

The organisational context documented in both cases suggests that institutional rules and procedural pathways may shape the conditions under which care is delivered and recorded. In Case 1, restricting the presence of family at critical phases of care reflect safety-oriented policies commonly implemented in high-dependency settings. In Case 2, access to specialised psychosocial support was documented as dependent on medical prescription, indicating adherence to established referral protocols. Within the disaster governance literature [5,28], such organisational arrangements are recognised as structural components that regulate decision-making authority, resource allocation, and professional scope of practice. While these governance mechanisms are designed to ensure coordination and safety during crisis response, they may also influence the visibility and timing of relational and psychosocial interventions. The present findings therefore illustrate how institutional norms and procedural frameworks can shape the configuration of humanised care in disaster-related hospitalisation, without implying that such structures inherently constitute barriers [10,21,29].

When the provision of essential care is compromised, negative impacts are felt not only by patients but also by families and informal caregivers, health professionals, and the health systems themselves. These consequences are particularly evident in highly complex and exceptional contexts such as disaster situations [10]. Essential care involves actions by the care team that respect and focus on the essential needs of a person, caregiver, or family to ensure their physical and psychosocial well-being [30]. They involve actions by the nurse that respect the essential needs of the person to ensure their physical activity and psychosocial well-being, and they are met through the development of a positive and trusting relationship with the person being cared for, as well as with the family/carers [10,29,30]. Care requires involvement, understanding of patients’ lives, and the reformulation of needs throughout care practice. Actions such as eating or drinking are not tasks, but moments of human involvement that preserve dignity and individuality.

The challenges that arise in contexts that hinder patient-centred care, particularly in disaster situations, are scarce resources that require priorities to be set. The complexity of fundamental care lies in multiple types of care that must be authentic and effective in a limited period of time [8,10]. Nurses must balance technical care needs with relational care, requiring specialised guidance to integrate assessment, intervention, and responsibility, as well as adapt care practices to disaster conditions [10,31].

Recognising the centrality of providing high-quality fundamental care, both through the adoption of risk mitigation activities and the importance of establishing trusting relationships, can create a common language across all clinical specialties and throughout a patient’s life. The focus on risk mitigation and positive outcomes of fundamental care enables health systems to ensure safe care delivery and support better recovery and well-being, even in exceptional situations such as disasters [10].

Considering these results, it is clear that the emerging needs of victims must be analysed in a multidimensional way, including the therapeutic relationship as a mediator of care, the integration of physical, psychosocial, and relational care, and the structural context, which defines the feasibility of interventions.

This study applies a contemporary theoretical framework (FoC) to nursing practices documented in 1997. While this retrospective interpretation enables a structured understanding of care delivery, it may also involve a degree of theoretical imposition. The framework was not available to practitioners at the time; therefore, the analysis should be understood as a contemporary reinterpretation rather than evidence of explicit theoretical alignment in the original context.

From an epistemological perspective, this approach can be conceptualised as a form of retrospective theoretical reframing or interpretive reconstruction, in which historical clinical material is examined through later-developed conceptual lenses. Such an approach generates analytical insight by sitting past practices within current disciplinary knowledge. Accordingly, the findings should be interpreted as theoretically informed reconstructions that illuminate patterns of care, while acknowledging the temporal distance between practice and development framework.

### 4.1. Implications for Practice and Future Research

Nursing in disaster situations should be understood as a dynamic and multidimensional process that addresses both the survival and long-term recovery needs. Nurses require specialised training that integrates technical emergency skills (e.g., stabilisation, ventilatory support, pain and shock management) with relational skills (e.g., communication, emotional support, and promotion of autonomy). The model based on the FoC framework, aligned with the outcome indicators developed in the ICNP and Health Policies, may contribute to greater standardisation of care, clearer prioritisation of patient needs, and improved continuity of quality care. While the present study does not provide empirical evidence regarding hospitalisation duration, structured integration of physical, psychosocial, and relational dimensions of care may support recovery processes and optimise care pathways in disaster-related contexts.

From a research perspective, multicentre and longitudinal studies are needed to explore how needs evolve across disaster phases and populations. Mixed methods are particularly relevant when clinical data are combined with the experiences of the survivors and their families. A convergent parallel design could integrate structured clinical documentation analysis with qualitative interviews of survivors and family members to triangulate documented care with lived experience. Alternatively, an explanatory sequential design could use quantitative analysis of nursing-sensitive indicators (e.g., documented psychosocial interventions, recovery markers, length of stay) followed by qualitative exploration of contextual and relational dimensions of care. However, studies on nurses’ interventions and their relational dimensions remain limited. Expanding methodologically rigorous research in this area is essential to validate and refine theoretically grounded care models.

### 4.2. Study Limitations

One limitation of this study was that only two cases were analysed. Nevertheless, the adopted in-depth approach made it possible to capture the complexity of the needs of disaster survivors. The heterogeneity of the cases analysed, one representing an acute critical trajectory and the other a prolonged hospitalisation, enhanced the analytical depth of the study by allowing examination of fundamental care across distinct phases of recovery. This variation supported theoretical replication and facilitated exploration of how physical, psychosocial, and relational dimensions of care may manifest differently according to clinical trajectory. However, this heterogeneity also limits the scope of analytic generalisation. The findings should therefore be interpreted as context-bound theoretical insights across all disaster settings or populations.

The use of retrospective clinical data may have limited access to the subjective dimension of the victims; however, it ensured documentary accuracy and fidelity to hospital reality, providing a consistent basis for future research that integrates participatory methodologies.

It is also important to recognise that the disaster analysed refers to a phenomenon with specific characteristics and does not cover all possible scenarios, namely other natural disasters such as earthquakes, floods, or fires, or events resulting from human action, such as technological accidents or armed conflicts. This limitation restricts the generalisation of the results, reinforcing the need for studies that consider different types of disasters.

Conversely, the integration of multiple theoretical frameworks, although challenging, strengthens the robustness and relevance of the analysis, opening perspectives for the development of integrative models capable of guiding nursing practice in disaster contexts. Thus, the identified limitations are seen as opportunities for furthering knowledge and promoting articulation between theory, practice, and health policies in response to catastrophic situations.

## 5. Conclusions

The Fundamentals of Care Framework has proven to be a particularly suitable theoretical framework for addressing disaster victims, as it supports nursing practices integrates the relational dimension, integration of care, and context of care. In a context marked by the urgency of clinical stabilisation and the high vulnerability of those affected, the FoC ensures that the technical response is not dissociated from the promotion of comfort, dignity, and family involvement. Thus, disaster nursing asserts itself as a multidimensional process, in which immediate stabilisation and overall recovery are articulated by integrative models, contributing to more humanised, consistent, and health-oriented care. In critical scenarios, interventions focused on physical fundamentals and safety were aimed at stabilisation and symptom control.

However, the findings suggest that more consistent outcomes emerge when these interventions are integrated with psychosocial and relational components, including emotional support, therapeutic communication, and family involvement, thereby promoting comfort, tranquillity, and adherence to care plans.

Simultaneously, the organisational context proved decisive for the implementation of fundamental care, underscoring how institutional policies and standards can either facilitate or constrain humanised, integrated responses—particularly regarding access to specialised psychosocial support and the involvement of family members.

In summary, disaster nursing should be conceptualised as a multidimensional process that integrates immediate stabilisation with holistic recovery. This process should be supported by integrative models, standardised language, and clearly defined indicators to ensure person-centred care, continuity, quality, and optimal health outcomes.

## Figures and Tables

**Table 1:** Eligibility criteria for case selection.

Category	Eligibility Criteria
Inclusion criteria	Victims directly affected by a disaster. Initial assessment in the Emergency Department as part of the hospital disaster response. Subsequent inpatient admission following Emergency Department observation. Availability of complete clinical and nursing records enabling the analysis of nursing needs and interventions. Purposeful case selection according to a multiple-case study design, allowing literal replication (similar expected patterns) or theoretical replication (predictable contrasting patterns).
Exclusion criteria	Disaster victims discharged directly from the Emergency Department without inpatient admission. Incomplete or absent nursing documentation prevented systematic analysis. Cases limited to the acute phase without sufficient longitudinal documentation.

**Table 2:** Domains, Diagnoses/Interventions, Expected Outcomes, and Assessment for Cases 1 and 2, according to ICNP [17].

Domain	Case	Nursing Diagnoses/Interventions	Expected Outcomes	Assessment
Respiratory	Case 1	Airway maintenance; chest drainage monitoring; breathing and saturation monitoring	Effective ventilation and adequate saturation	Respiratory stabilisation confirmed in clinical records
Cardiovascular	Case 1	Fluid therapy via CVC; administration of diuretic; monitoring of perfusion	Effective perfusion and haemodynamic stability	Haemodynamic stability achieved
Pain/Trauma	Case 1	Analgesia (morphine, paracetamol); pain monitoring	Pain reduction and increased comfort	Pain controlled after analgesia
	Case 2	Multimodal analgesia (nolotil IV/IM, paracetamol); response monitoring	Effective pain control and increased comfort	Pain controlled after therapeutic adjustment
Integument/Safety	Case 1	Prevention of pressure injuries; CVC monitoring	Intact skin and no complications	Complications avoided
	Case 2	Skin care; application of gel cushion for pressure relief	Intact skin and prevention of pressure ulcers	No complications recorded
Psychological	Case 1	Emotional support; presence of family member; psychological counselling	Reduction in anxiety	Less crying and greater tranquillity
	Case 2	Emotional assessment; psychiatric support	Stabilisation of mood	Partial improvement observed
Mobility/Musculoskeletal	Case 2	Skeletal traction monitoring; immobilisation; pressure ulcer prevention	Maintenance of function and prevention of complications	No functional deterioration
Therapeutic management	Case 2	Blood/plasma transfusions; medication adjustments	Safe therapeutic compliance	Successful transfusions

**Table 3:** Identification of Fundamental Care based on the Relationship Dimension of the Fundamental Care Framework by Kitson, Conroy [9].

Dimension 1.	Fundamental Care	Case 1	Case 2
Relationship	Developing and maintaining trust	On the last day (5th) of her stay in the ICU, she “calls the nurses her godmothers.”	Not documented
	Focus on the patient/person being cared for	“Maintains spontaneous, mixed, medium amplitude and regular breathing”; “continues to mobilise all limbs (…) preferring left lateral decubitus”; “spent the shift sleeping calmly”	“Patient spent the shift awake, calm and cooperative”; “has pressure areas on the coccyx and buttocks”; “began clamping the algalia, which has been controlled”; “patient very dissatisfied with food lately, having food brought from the cafeteria for him, eating well at dinner”
	Anticipating the needs of the patient/person	If he becomes agitated and wants the company of a family member, call the lady.”	“still has no sensation in the lower limbs”
	Get to know the patient/person and the best way to care for them	“seems to refuse contact with the outside world”; “asked to draw the curtain”	“The patient remains in the same condition…”
	Assess the quality, progress and results of the relationship	Day 13—“at the beginning of the shift, she was (…) talking to us and seemed in good spirits”	“ate well at dinner, received a visit from a family member and reacted well”

**Table 4:** Identification of Fundamental Care based on the Care Integration Dimension of the Fundamental Care Framework of Kitson, Conroy [9].

Dimension 2. Care Integration	Fundamental Care	Case 1	Case 2
Physical fundamentals of care (needs and outcomes of the care recipient)	Personal hygiene (including oral/mouth care) and dressing	“Dry oral mucosa; patient requested water after oral moistening.” “dry and chapped lips lubricated with Vaseline”	Not documented
	Hygiene needs	“Cooperated little in providing hygiene care, refusing to wash her face and hands by herself”; “Hygiene care in the bathroom, reacting well”	“Hygiene and comfort care provided”
	Eating and drinking	“At snack time, she ate ½ a yoghurt and at dinner she refused to eat, drinking only ½ a glass of milk”; “At lunch, she ate all of her soup, a sausage and a little egg”	“At dinner, she refused the second course”; “Ate poorly, only soup and fruit.” “Refused dinner due to feeling unwell.” “Ate reasonably well”.
	Rest and sleep Mobility	“Restless sleep, motor restlessness, unable to find a comfortable position, getting up, attempting to pull out tubes; periods of crying, wanting his mother”; “Had some periods of light sleep”; “Spent the shift sleeping calmly”	“Slept for the remainder of the shift, alternating between sleep and wakefulness.” “at the beginning of the shift had difficulty falling asleep, took a Valium 5 tablet, which is prescribed in SOS situations”; “patient reported no complaints, slept well”; “slept through the night”
	Comfort (e.g., pain control, easy breathing, temperature control)	“Spontaneous breathing with regular and mixed respiration”; “Reported abdominal pain, administered analgesia”; “Was apyretic”	“patient subfebrile”; “axillary temperature at 12 noon, 37.5 °C, no antipyretic administered”; “at 8 pm had a temperature of 38.4 °C, administered 1 g of paracetamol”; “ reported pain, administered 1 capsule of nolotil at around 11 am”; “At 6 pm, the patient complained of pain in the lower limb and was given 1 capsule of nolotil, which was not very effective. For this reason, an intramuscular nolotil formula was administered at 10 pm, which was effective.”
	Safety (e.g., risk assessment and management, infection prevention, minimisation of complications)	“chest drainage not functioning”; “skin near the central venous catheter insertion site shows slight redness”; “heart tracing when asleep shows tendency to bradycardia”; “pressure area on the inner side of the right foot, pad applied”	“Gel cushion applied, maintains decreased sensitivity in left leg”; “Pressure area on coccyx and buttock”; “Lower limb with skeletal traction, extremities without changes”
	Medication management	“There was a change in therapy”; “low urine volume, 2.5 mg of furosemide was administered, which was effective”; “reported pain in the right hemithorax, administered morphine as prescribed, fell asleep again”; “When feeding begins, medication will be switched to oral administration”; “complied with medication”; “reasonable urinary volumes without the need for diuretics”	“Therapy was changed and tests were requested for tomorrow, as well as one unit of red blood cells and two units of fresh frozen plasma, which the patient has already received without incident”; “Complied with the proposed therapy”; “An intravenous formula of nolotil diluted in 100 cc of SF was administered 1 h and 30 min ago and was effective”
Psychosocial foundations of care (needs and outcomes of the care recipient)	Communication (verbal and non-verbal)	“absent gaze”; “when family members visited, she responded through gestures regarding people she recognized, but did not speak to them”; “refuses contact with the outside world, asking for the curtain to be drawn”; “when questioned, she responds after insistence and with some disinterest”; on the 9th day, “she received a visit from the psychologist, who spoke with her. From then on, she cried intensely”; “she said she was afraid of being alone”; 15th day “awake and talking to us, appearing in good spirits”	“She appears sad about her situation”; “(…) quite discouraged, trying to react”; “Dr. Requested psychiatric support”; “appears depressed”
	Stay involved and informed	Not documented	Not documented
	Privacy	“asked to close the curtains”	Not documented
	Dignity	Not documented	Not documented
	Respect	Not documented	Not documented
	Education and information	“The family has been informed to schedule an ophthalmology appointment in December (…) Upon leaving, she contacted the social worker (…) The family member spoke with the psychologist, who is responsible for notifying them of the next psychiatry appointment.”	“There was a change in therapy, all medication was suspended, and after a telephone call with Dr. X, the patient should continue taking multivitamins three times a day.”
	Emotional well-being	“At around 7:30 A.M., she woke up crying and calling for her family member, who was contacted by telephone (…) with her presence, she stopped crying.”	“The patient was in a very good mood throughout the shift and remained upright. She walked around with the walking frame.”
	Having values and beliefs that are considered and respected	Not documented	Not documented
Relational foundations of care (actions of the care provider)	Active listening	Not documented	Not documented
	Being empathetic	“She cried for a long time, hugging her family member and saying she did not want her to leave. After a while, she fell asleep peacefully”; “If she becomes agitated and wants her family member’s company, call the lady.”	Not documented
	Getting involved with patients	Not documented	Not documented
	Being compassionate	Not documented	Not documented
	Be present and with the sick	Not documented	Not documented
	Supporting and involving families and carers	“Crying and calling for her relative, she was contacted by telephone”; “Spent the shift in the company of family members”; “Please give the dinner request to the family member”; “Was playing games with the family member”	Not documented
	Helping patients cope	Not documented	Not documented
	Working with patients to set, achieve and evaluate the progress of goals	Not documented	Not documented
	Helping patients to remain calm	Not documented	Not documented

**Table 5:** Identification of Fundamental Care based on the Context of Care Dimension of the Fundamental Care Framework by Kitson, Conroy [9].

Dimension 3.	Fundamental Care	Case 1	Case 2
Care Context	Includes policy and system-level factors that impact the caregiver’s ability to develop a relationship with the person being cared for and to meet their fundamental needs in an integrated manner	Institutional policy of not allowing family members to stay during full-time hospitalisation, especially at night. Requirement for a medical prescription for psychological support.	Not documented

## Data Availability

Due to privacy, the datasets used and analysed in this study are available from the corresponding author on reasonable request.

## Public Involvement Statement

No public involvement in any aspect of this research.

## Guidelines and Standards Statement

This manuscript was drafted following the Standards for Reporting Qualitative Research (SRQR) for qualitative research.

## References

[1] Tussing T.E., Chesnick H., Jackson A. (2022). Disaster Preparedness Keeping Nursing Staff and Students at the Ready. Nurs. Clin. N. Am..

[2] (CRED) CfRotEoD (2025). 2024 Disasters in Numbers.

[3] (UNDRR) UNOfDRR (2024). The Science—Policy—Society Ecosystem for Disaster Risk Reduction: Words into Action.

[4] (UNISDR) UNOfDRR, Reduction UNOfDR (2015). Sendai Framework for Disaster Risk Reduction 2015–2030.

[5] (ICN) nCoN (2019). Core Competencies in Disaster Nursing Version 2.0.

[6] Veenema T.G. (2018). Disaster Nursing and Emergency Preparedness for Chemical, Biological, and Radiological Terrorism and Other Hazards.

[7] Khorram-Manesh A., Mani Z. (2025). Navigating the chaos: A scoping review of gaps in disaster nursing and a roadmap for the future. BMC Nurs..

[8] (ICN) ICoN (2022). Core Competencies in Disaster Nursing: Competencies for Nurses Involved in Emergency Medical Teams (Level III).

[9] Kitson A., Conroy T., Kuluski K., Locock L., Lyons R. (2013). Reclaiming and Redefining the Fundamentals of Care: Nursing’s Response to Meeting Patients’ Basic Human Needs.

[10] Kitson A., Carr D., Feo R., Conroy T., Jeffs L. (2025). The ILC Maine statement: Time for the fundamental care [r]evolution. J. Adv. Nurs..

[11] Kitson A. (2020). Fundamentals of care: Methodologies, metrics and mobilisation. J. Clin. Nurs..

[12] Kitson A., Feo R., Lawless M., Arciuli J., Clark R., Golley R., Lange B., Ratcliffe J., Robinson S. (2022). Towards a unifying caring life-course theory for better self-care and caring solutions: A discussion paper. J. Adv. Nurs..

[13] Yin R.K. (2018). Case Study Research and Applications: Design and Methods.

[14] Network T.E. (2025). The SRQR Reporting Checklist.

[15] Lourenço L. (2004). Riscos Naturais e Proteção do Ambiente.

[16] Oficial J., SRPCBA (2019). Plano Regional de Emergência de Proteção Civil dos Açores.

[17] (ICN) ICoN (2017). International Classification for Nursing Practice (ICNP^®^) Catalogue.

[18] Denzin N.K., Lincoln Y.S. (2018). The SAGE Handbook of Qualitative Research.

[19] Rezaei S.A., Abdi A., Akbari F., Moradi K. (2020). Nurses’ professional competences in providing care to the injured in earthquake: A qualitative study. J. Educ. Health Promot..

[20] Mert I.S., Koksal K. (2025). Unveiling the heart of disaster nursing: A qualitative study on motivations, challenges, and lessons from the devastating 2023 Turkey earthquakes. Int. Nurs. Rev..

[21] Pene B.J., Aspinall C., Komene E., Slark J., Gott M., Robinson J., Parr J.M. (2025). The Fundamentals of Care in Practice: A Qualitative Contextual Inquiry. Nurs. Inq..

[22] Molina-Mula J., Gallo-Estrada J. (2020). Impact of Nurse-Patient Relationship on Quality of Care and Patient Autonomy in Decision-Making. Int. J. Environ. Res. Public Health.

[23] Pierre S.D., Ramos M.C., Shimizu H.E. (2024). What Are the Best Practices for Nursing Care during an Earthquake? A Scoping Review. Int. J. Environ. Res. Public Health.

[24] Segev R., Suliman M., Gorodetzer R., Zukin L., Spitz A. (2025). Nursing roles in disaster zones: Experiences and lessons from Turkey’s 2023 earthquakes. Int. Nurs. Rev..

[25] Seren A.K.H., Dikeç G. (2023). The earthquakes in Turkey and their effects on nursing and community health. Int. Nurs. Rev..

[26] Kaya S.S., Erdogan E.G. (2025). Being a nurse during an earthquake that affected ten provinces: A qualitative study on experiences and expectations. Int. Nurs. Rev..

[27] Su Y., Wu X.V., Ogawa N., Yuki M., Hu Y., Yang Y. (2022). Nursing skills required across natural and man-made disasters: A scoping review. J. Adv. Nurs..

[28] Firouzkouhi M., Kako M., Abdollahimohammad A., Balouchi A., Farzi J. (2021). Nurses’ Roles in Nursing Disaster Model: A Systematic Scoping Review. Iran. J. Public Health.

[29] Kitson A.L., Conroy T., Jeffs L., Carr D., Huisman-Dewaal G.J., Muntlin A., Jangland E., Grønkjær M., Parr J. (2023). ‘No more heroes’: The ILC Oxford Statement on fundamental care in times of crises. J. Adv. Nurs..

[30] Feo R., Conroy T., Jangland E., Athlin A.M., Brovall M., Parr J., Blomberg K., Kitson A. (2018). Towards a standardised definition for fundamental care: A modified Delphi study. J. Clin. Nurs..

[31] Jeffs L., Merkley J., Ronald K., Newton G., Yang L., Gray C.S. (2023). Can fundamental care be advanced using the science of care framework?. J. Adv. Nurs..

